# Why Medical Students Are Crucial to the Future of Research in South Asia

**DOI:** 10.1371/journal.pmed.0020322

**Published:** 2005-11-29

**Authors:** Fawad Aslam, Murtaza Shakir, Muhammad Ahad Qayyum

## Abstract

One long-term strategy for promoting health research in developing countries is to target medical students early in their careers.

Health research is essential to improving health care [1]. Unfortunately, health research has a low priority in the developing world. In all disciplines of science and technology, India and Pakistan combined have 208 researchers per million citizens [2,3], as compared to 4,526 researchers per million citizens in the United States [4]. The published research output from South Asia is small—South Asian health researchers accounted for only 1.2% of all papers within the Institute for Scientific Information database from 1992–2001 [5]. Developing countries must therefore enhance their research capacity to efficiently address the growing burden of both communicable and non-communicable diseases [6].

## Engaging Medical Students in Health Research

One long-term strategy for promoting health research is to target medical students early in their careers. Most of the research to date on the effectiveness of such a strategy has been done in Western settings. This research has shown that research experience as a medical student is strongly associated with postgraduate research involvement [7,8].

Student research can also contribute to the published output of an institution. In Germany, for example, medical students authored 28% of the publications of one institution, including first authorship in 7.8% of papers [9]. Nothing can be more motivating for a student than to get published.

Even if the experience of doing research as a student does not lead to a later career in academic medicine, research experience can help improve students' skills in searching and critically appraising the medical literature, independent learning, and writing research papers [10,11]. Such exposure to research as a student can also help to identify future careers, establish important contacts, and secure better residency positions. Given the many benefits of doing a research project as a student, not surprisingly, 97% of students considered research as a useful alternative to electives [11].

Student research is not without its problems. Good mentorship, for example, is a vital component of effective student research, and inadequate mentoring can lead to discontentment with research. Other problems include lack of time, neglect of routine studies and deterioration of clinical skills due to more time being spent on research activities, and inadequate project management [12]. The perceived competitiveness and greater demands of a career in academic medicine and lower salary compared to private practice may deter students from research. Another concern is that students may work simply as junior laborers with no role in designing the research or in critical thinking during the research process. For example, they may be told to review, say, 200 charts, and hand over the data to the principal investigator. Those who criticize medical student research would also argue that student papers are rarely cited, and thus are of limited utility.

## Student Research in South Asia

Student research is dependent on national research activity. Since there is still only a limited research infrastructure in many developing countries, this means that opportunities for medical student research are limited. Research is not considered a part of the medical curriculum in many of these countries.

In one Indian study, for example, 91% of interns reported no research experience in medical school [13]. Thus, students in India rarely get exposed to research at this crucial stage in their academic development when such exposure could encourage further research after qualification. Faculty across South Asia themselves seldom engage in research owing to inadequate training in research, lack of incentives, work overload, poor pay, and minimal research demand in clinical practice from patients, fellow physicians, and policymakers. Consequently, students are deprived of mentors and role models. Medical training in general in South Asia does not emphasize the importance of research to medical practice. More than two-thirds of the postgraduate trainees at one Pakistani institution, for example, reported reading scientific journals only once in six months or more [14].

## Taking Action

**Figure 1:**
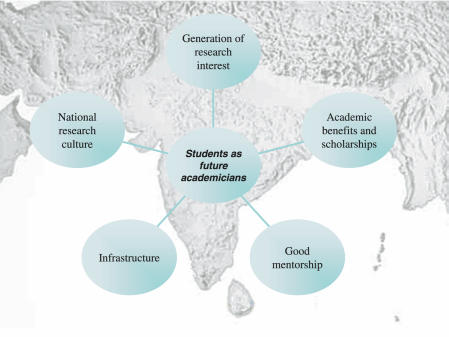
Factors That Can Help Encourage South Asian Medical Students to Choose a Career in Academic Medicine

What can be done to increase medical students' involvement in research in South Asia (Figure 1)? First and foremost, the research infrastructure needs extensive improvement, and the meager funding for research must be boosted, so that there will be a healthier research culture in which students can participate. There also need to be effective international agreements to halt the “brain drain” of academic clinicians from low-income to high-income countries [15], since this migration robs medical students of role models.

In addition, students need to be “sensitized” to research—that is, they should be made aware of why research is so crucial to health care. The medical curriculum must begin to incorporate and emphasize evidence-based medicine. To stimulate students' interest in research, we believe that they should undertake a mandatory course on research skills, along with a compulsory community project. Such projects undertaken in the primary-care setting will allow students to execute all the steps of a research project, from conception to final report writing, thereby narrowing the gap between theory and practice. Elective slots should be available to those students who are interested in pursuing further research. Young researchers need to be encouraged, for example, by being awarded scholarships. Prior research experience should be seen as valuable when recruiting postgraduate trainees. Only when students are sensitized in this way—and provided that they also have sufficient flexibility and security to pursue a career in academic medicine—will they choose career paths involving research.

## Conclusion

At our own medical schools, we believe that medical students are becoming more enthusiastic about getting involved in research, which is encouraging. Some efforts to promote student research are already underway in South Asia. For example, student conferences and research workshops are being held in major cities of Pakistan, and some medical journals, including the *Journal of Postgraduate Medicine* (India) and the *Journal of the College of Physicians and Surgeons of Pakistan*, have introduced student sections. The students at Aga Khan University in Pakistan, which has a well-established research infrastructure, have won awards for their projects at international student conferences and have published widely in indexed journals. Given the right amount of support, medical students' interest in research can be successfully nurtured.
